# When attitudes and beliefs get in the way of shared decision‐making: A mediation analysis of participation preference

**DOI:** 10.1111/hex.13699

**Published:** 2023-01-13

**Authors:** Björn Büdenbender, Anja K. Köther, Britta Grüne, Maurice S. Michel, Maximilian C. Kriegmair, Georg W. Alpers

**Affiliations:** ^1^ Department of Psychology, School of Social Sciences University of Mannheim Mannheim Germany; ^2^ Department of Urology and Urosurgery, University Medical Center Mannheim University of Heidelberg Mannheim Germany

**Keywords:** patient attitudes, patient‐centred healthcare, patient participation, patient preferences, patient‐reported barriers, shared decision‐making

## Abstract

**Introduction:**

Certain sociodemographic characteristics (e.g., older age) have previously been identified as barriers to patients' participation preference in shared decision‐making (SDM). We aim to demonstrate that this relationship is mediated by the perceived power imbalance that manifests itself in patients' negative attitudes and beliefs about their role in decision‐making.

**Methods:**

We recruited a large sample (*N* = 434) of outpatients with a range of urological diagnoses (42.2% urooncological). Before the medical consultation at a university hospital, patients completed the Patients' Attitudes and Beliefs Scale and the Autonomy Preference Index. We evaluated attitudes as a mediator between sociodemographic factors and participation preference in a path model.

**Results:**

We replicated associations between relevant sociodemographic factors and participation preference. Importantly, attitudes and beliefs about one's own role as a patient mediated this relationship. The mediation path model explained a substantial proportion of the variance in participation preference (27.8%). Participation preferences and attitudes did not differ for oncological and nononcological patients.

**Conclusion:**

Patients' attitudes and beliefs about their role determine whether they are willing to participate in medical decision‐making. Thus, inviting patients to participate in SDM should encompass an assessment of their attitudes and beliefs. Importantly, negative attitudes may be accessible to change. Unlike stable sociodemographic characteristics, such values are promising targets for interventions to foster more active participation in SDM.

**Patient or Public Contribution:**

This study was part of a larger project on implementing SDM in urological practice. Several stakeholders were involved in the design, planning and conduction of this study, for example, three authors are practising urologists, and three are psychologists with experience in patient care. In addition, the survey was piloted with patients, and their feedback was integrated into the questionnaire. The data presented in this study is based on patients' responses. Results may help to empower our patients.

## 1 INTRODUCTION

### 1.1 Models of patient–provider relationship

In recent years there has been a fundamental societal shift in how we think about patients' role in treatment and decision‐making.^1^ Moving away from the outdated paternalistic decision‐making model, in which the patient typically has a passive role with little autonomy and responsibility, the gold standard is shared decision‐making (SDM).

SDM is an interactive process in which patients and healthcare providers share information about available treatment options, risks and benefits and personal preferences for specific treatment options. SDM entails decision‐support counselling (deliberation of options) and joined implementation of the decision.^2^, ^3^ Thus, SDM is neither paternalistic (i.e., the physician decides alone) nor does it leave the patient alone.^2^, ^4^, ^5^ A recent integrative model of SDM^4^ defined the nine most essential elements necessary for SDM: define/explain the problem, present options, discuss pros/cons, patient values/preferences, discuss patient ability/self‐efficacy, provider recommendation, check/clarify understanding, make or explicitly defer the decision, arrange follow‐up.^4^, ^6^ SDM has been recommended as an ethical imperative and is considered a hallmark of patient‐centred care.^7^, ^8^, ^9^ The ideal SDM process^6^ fosters a patient's right to self‐determination and places value on his or her voice. Thus, enabling the delivery of truly individualized care,^10^ which is at the heart of patient‐centred care.

Although there appears to be a widespread consensus among stakeholders (e.g., patients, policymakers and providers) on the relevance of SDM and despite the increasing international efforts to promote SDM, implementation has proven to be difficult and slow.^8^, ^11^ As part of a larger project to promote SDM in urological practice, we, thus, set out to investigate the obstacles that prevent patients from participating in SDM.

### 1.2 Relevance of participation preference

SDM has quantifiable benefits; the involvement of patients in treatment decisions is, for example, associated with better treatment adherence,^12^, ^13^, ^14^ more accurate knowledge about their condition and recommended treatments^15^ and less decisional conflict.^16^ Ultimately, this leads to higher satisfaction among patients^16^, ^17^ and physicians^7^ alike. Some studies even report a higher quality of life.^18^, ^19^ Beyond these sizable effects, SDM is also considered the ethical imperative for treatment decisions as it fosters patients' right to self‐determination.^9^, ^20^ Consequently, most modern healthcare systems have implemented legislation to facilitate SDM.^8^

Despite these benefits, not all patients seek participation in decision‐making to the same extent.^15^, ^21^, ^22^, ^23^ Although a general trend towards a stronger preference to participate has been observed over the last decades,^24^ about a quarter of patients (Md = 27% in a review of 31 studies^23^) express a low desire for autonomy. Research has recently started to identify reasons for this.

Existing research reported lower participation preference in patients with certain sociodemographic characteristics. In particular, older patients, less educated individuals and men^18^, ^25^, ^26^, ^27^ express lower interest in active participation. However, a comprehensive review indicates that the exact nature and mechanisms involved in such sociodemographic variables' on participation preference remain to be specified in cancer patients.^28^ Thus, one goal of this study was to investigate the association of sociodemographic variables on the participation preference of urooncological patients.

### 1.3 Power imbalance as a potential barrier

It has recently been suggested that a seemingly low desire for patient autonomy may be due to a perceived lack of personal ability to participate rather than a genuine lack of actual desire.^29^ In particular, the power imbalance (i.e., the perceived permission to participate) in the physician–patient relationship has been hypothesized to be a critical determinant of patients' ability to participate in medical decision‐making. Patients often underestimate the value of their contributions to the consultation, that is, their ability to understand medical information and the importance of their personal preferences,^30^, ^31^ which contributes to an increased feeling of a power imbalance. Consequently, these individuals tend to delegate decisions to medical experts. The asymmetry in the physician–patient relationship is deepened if patients feel dependent on their physician's benevolence.^32^

For example, patients may fear being labelled a difficult patient and the negative impact that such a label could have on their care.^33^, ^34^, ^35^ Thus, they refrain from asking too many questions or asserting their interests, such as actively participating in decision‐making. All too often, the normative belief prevails that a ‘good’ patient is characterized by conformity and passivity, which may overshadow patients' desire for autonomy.^36^ Several studies report that even when patients witness a potentially harmful and preventable event during their care (e.g., a lack of hygiene measures), a majority of (cancer) patients opt not to speak up or assert their interest.^37^, ^38^, ^39^ This phenomenon has previously been labelled as white‐coat silence,^40^ and it is closely related to patients' attitudes regarding their role in decision‐making and beliefs about what constitutes a ‘good’ patient. This effect is particularly pronounced when the stakes are high, as is the case for life‐threatening illnesses due to the increased dependence on the physician. In extreme cases, patients may even describe themselves as being hostages to the situation.^41^

Considering patients' attitudes and beliefs about their role thus appears essential in optimized SDM. However, these patient variables are not routinely assessed even when SDM is carefully implemented. Therefore, it is crucial to investigate the potential barriers that attitudes and beliefs impose and may curtail patients' potential to participate in medical consultation actively. Thus, we seek to analyse the relationship between patients' attitudes and beliefs about their role and their participation preference.

### 1.4 Attitudes' and beliefs' influence on behaviour

The relevance of attitudes and beliefs for behaviour preference (i.e., participation preference) is well‐founded in psychological theories such as the theory of planned behaviour.^42^ From this perspective, a particular behaviour (e.g., participation in decision‐making) is preceded by an intention (or behavioural preference).^43^ In the SDM context, this implies that the act of participating requires a certain participation preference. The theory of planned behaviour postulates three important determinants for such a specific behavioural preference: attitudes towards the behaviour, control beliefs and normative beliefs. We, therefore, examine whether the relationship between patient characteristics and participation preference is mediated by attitudes towards the behaviour (participating in decision‐making), control beliefs (presence of factors that facilitate or impede participation, e.g., lack of knowledge), as well as normative beliefs (what defines a ‘good’ patient).

Importantly, prior work has identified the potential of targeting attitudes and beliefs regarding SDM in healthcare professionals to foster change in behaviour intentions and behaviour itself.^44^ However, there are no investigations on the role of attitudes and beliefs in patients.

### 1.5 Current study: SDM in urooncology

SDM has been used and evaluated in a diverse range of medical subdisciplines. A recent review by the American Urological Association (AUA) concluded that it is woefully underutilized^45^ in the urological domain. However, SDM is increasingly requested in treatment guidelines and has proven to positively impact urological patients' knowledge, decisional conflict and quality of life.^45^

Thus, in the context of a larger project to foster SDM in general urological practice, we set out to study barriers that impede urological patients' desire and ability to participate. Integration of SDM and engagement of patients is especially relevant in urology because patients in urology and urooncology often face very difficult and highly preference‐sensitive decisions,^46^, ^47^ in which patient participation can have favourable effects. For instance, early‐stage prostate cancer treatment demands patients and physicians to collaboratively decide among many treatment choices in equipoise, such as active surveillance, surgery and various forms of radiation treatments.^47^, ^48^ Regarding the participation preference of patients in urology, there have been a few studies on prostate cancer. In this patient group, there is a relatively high preference for participation compared to other tumour entities^22^, ^23^; however, around 10%–15% of patients report a low participation preference.^15^, ^23^, ^49^

While previous research has typically addressed the influence of stable sociodemographic variables^18^, ^25^, ^26^, ^27^, ^28^ on participation preference, we also aimed to gather information on potential mediators. We hypothesize that the previously reported association between certain patient characteristics and a low preference for participation^18^, ^26^, ^50^ is mediated by a perceived power imbalance, which we expect to be reflected in patients' attitudes towards active participation, control beliefs and normative beliefs about the patient role.

With this research, we aim to identify potential targets for interventions that may help to empower patients who are reluctant to engage in SDM. Unlike stable sociodemographic characteristics, attitudes and beliefs are more accessible to change^43^ and, therefore, may be ideal targets for structured interventions to help foster patient engagement and increase patient participation in the SDM process. For example, rather than assuming that older cohorts will most likely not be as open to active participation in decision‐making, knowing more about the mechanisms in this process may encourage clinicians to pay close attention to their attitudes and beliefs.

## 2 METHODS

### 2.1 Participants

We recruited a sample of *N* = 468 consecutive patients who had a scheduled appointment or presented unscheduled with urgent symptoms at the outpatient clinic of the Department of Urology and Urosurgery at University Medical Center Mannheim, Germany. We specified the following inclusion criteria: at least 18 years old, adequate proficiency in German and the ability to give informed consent. We excluded 3 patients with obviously invalid responses (i.e., only marked the middle response category on all questionnaires) and 15 patients (3.2%) with more than 50% missing data. In addition, 16 patients (3.4%) dropped out after giving informed consent.

Excluded patients (*n* = 31, 6.6%) were compared to those in the final sample (*N* = 434) by Pearson *χ* ^2^‐tests. When necessary (a cell with an expected cell count below one), we calculated Fisher's exact test.^51^ There were no differences between excluded patients and the final sample regarding the type of appointment (appointment or unscheduled), diagnosis and occupational status (all *p*'s > .117). However, more excluded patients lived alone (*χ* ^2^(1) = 20.56, *p* ≤ .001, φ = −0.21), were female (*χ* ^2^(1) = 6.761, *p* = .017, φ = 0.121) and had a lower level of education (no university degree) (*χ* ^2^(1) = 6.421, *p* = .011, φ = −0.117). All effect sizes are considered small.^52^

The final sample of *N* = 434 was between 19 and 89 years old (*M* = 62.5, SD = 13.5), primarily male (87.3%) and with a scheduled appointment (88.7%). Patients had a variety of urological diagnoses (42.2% urooncological).

### 2.2 Data collection and measures

Patients were approached before their consultation with a urologist by a nurse or a research assistant; they were informed about the protocol. After they signed informed consent, patients provided sociodemographic characteristics and filled in a set of self‐report questionnaires, including the Autonomy Preference Index (API)^53^, ^54^ to assess participation preference and the Patients' Attitudes and Beliefs Scale (PABS).^55^ Additional questionnaire data, for example, on patients' anxiety and depression, are reported elsewhere.^56^, ^57^ Medical diagnoses were retrieved from patients' electronic health records.

#### 2.2.1 Demographic and medical variables

We collected standard sociodemographic information about patients' age, gender, level of education, marital status and living arrangement. We dichotomized the following variables: education (0 = without and 1 = with a university degree), living arrangement (0 = living alone or 1 =  with others) and occupation (0 = currently unemployed/retired or 1 = currently either employed/in training). In addition, medical diagnoses were categorized as (0) nononcological versus (1) oncological. For consistency, we applied dichotomization cut‐offs established in previous studies.^56^, ^57^

#### 2.2.2 Participation preference and intention to participate

The primary outcome of our study is patients' behaviour preference for participating in medical decision‐making. Patients' preferences for involvement were measured with the German version of the API.^53^, ^54^ The measure consists of two subscales: decision‐making (API‐dm) and information‐seeking (API‐is). The German API‐dm subscale has four items, which assess generic participation preference, and the API‐is contains seven items, which assess information seeking. On both subscales, a linear transformation (0–100) is applied.^58^ Higher scores indicate a stronger desire for autonomy. We report descriptive statistics for the API‐is subscale. However, because there were high ceiling effects and little variance, the subscale was not used in further analyses (compare, e.g., Benbassat and colleagues^27^, ^59^, ^60^). The primary outcome of our study, patients' participation preference, was thus assessed with the API‐dm subscale. The API‐dm is often used on its own to assess participation preference, and it has been validated in various languages (e.g., German) and settings.^24^, ^61^ Internal consistency for the scale was good *α* = .85.^58^

In addition, we generated an item to ask patients about their intention to participate: ‘Regarding your upcoming consultation: Do you intend to participate in decision‐making?’. The item was rated on a 6‐point Likert Scale ranging from (‘Absolutely not’ to ‘Absolutely yes’).

#### 2.2.3 Attitudes and beliefs

We assessed patients' attitudes and beliefs about their role in shared treatment decisions using the PABS.^55^ The PABS was translated to German by the first author and independently back‐translated by a bilingual speaker. There were no discrepancies between the two versions. The German version can be obtained in Supporting Information: Appendix A. The PABS consists of 12 items on 2 subscales: 7 items for positive attitudes (e.g., ‘I have the right to make my own medical decisions, after all, it's my life’) and 5 for negative attitudes (e.g., ‘It would offend my doctor if I were to make my own decision(s)’). Patients rate their agreement to the items on a 5‐point Likert scale ranging from ‘do not agree at all’ to ‘fully agree’. The original English items were generated by multiple patient focus groups, strengthening the content validity of the scale.^55^ Internal consistency for the negative and positive subscale was acceptable with *α* = .71 and .73, respectively.^55^ Total scores were calculated by summing up all items on the respective subscales (positive attitudes and beliefs and negative attitudes and beliefs) and linearly transforming them to range from 0 to 100.

### 2.3 Statistical analyses

A drop‐out analysis was conducted to assess systematic differences between completers and noncompleters (see Section 2.1). We compared patient characteristics for patients with urooncological versus nonurooncological diagnoses with *χ* ^2^‐tests. Furthermore, patient subgroups were compared (independent sample *t*‐test) regarding participation preference, attitudes and beliefs. Univariate descriptive statistics are reported for participation preference, attitudes and beliefs and intention to participate, along with the Pearson correlation coefficients for the bivariate associations between these variables. The association between nominal scaled sample characteristics (e.g., gender) and the dependent variable participation preference were analysed with the correlation ratio *η*.

The expected mediation (i.e., sociodemographic variables—attitudes and beliefs—participation preference) was assessed with a path model (based on structural equation modelling). We checked the multivariate normality assumption between all included endogenous variables with the MVN R‐package.^62^ The path analysis was calculated with the lavaan R‐package^63^ using R.^64^ The complete list of the R‐packages is listed in Supporting Information: Appendix B. All other analyses were conducted in IBM SPSS, Version 27.0. Significance for all tests was set at *α* = .05. Where applicable, we report effect sizes; interpretation is based on Cohen's taxonomy.^52^

## 3 RESULTS

### 3.1 Sample characteristics

The final sample (*N* = 434) was between 19 and 89 years old (*M* = 62.5, SD = 13.5), mostly male (87.3%), and German nationals (97.5%). The majority of patients had scheduled appointments (88.7%). Patients had a range of urological diagnoses (42.2% urooncological: 27.2% prostate cancer, 8.1% bladder cancer, 6.3% other urooncological tumour entities, 0.7% missing information). Further sociodemographic characteristics are reported in Table 1.

**Table 1:** Sample characteristics and comparison of oncological with nononcological patients

Characteristic	Full sample (*N* = 434)		Oncological patients (*n* = 183)		Nononcological patients (*n* = 251)	
	*n*	%	*n*	%	*n*	%
Gender^a^						
Male	378	87.1	169	92.3	209	83.6
Female	55	12.7	14	7.7	41	16.4
Nationality						
German	423	97.5	181	98.9	242	96.4
Other	11	2.5	2	1.1	9	3.6
Living arrangement						
Alone	93	21.4	35	19.1	58	23.1
With others	341	78.6	148	80.9	193	76.9
Higher education						
No	277	63.8	115	62.8	162	64.5
Yes	157	36.2	68	37.2	89	35.5
Occupation status						
Unemployed	11	2.5	3	1.7	8	3.2
Apprenticeship/training	24	5.5	7	3.9	17	6.8
Employed	173	39.9	56	31.1	117	46.8
Retired	222	51.2	114	63.3	108	43.2

*Note*: Diverging numbers of patients from the total sample size are due to missing values.

^a^ The option ‘divers’ was available but chosen by no patient.

The subgroup of oncological patients (*n* = 183) contained significantly more males (*χ* ^2^(1) = 7.295, *p* = .007, φ = −0.13), more were retired (*χ* ^2^(3) = 17.17, *p* = .001, φ = 0.2), and they were on average older (*M* = 66.7, SD = 11.2) than the nononcological patients (*M* = 59.4, SD = 14.3), *t*(429.497), *p* ≤ .001, with a medium effect size of Hedge's *g*_*s*_ = −0.559.^52^ See Table 1 for further comparison of both subgroups. However, the two diagnostic groups did not differ in their attitudes and beliefs, intention to participate or the primary outcome participation preference (all *p*'s ≥ .104).

### 3.2 Patients' participation preference and associated variables

Overall, most patients wanted to participate to some degree in decision‐making (*M* = 44.8, SD = 26.5). Descriptively patients reported higher scores for positive attitudes (*M* = 64.9, SD = 14.4) than for negative attitudes (*M* = 55.8, SD = 12.8). Univariate descriptive statistics of metric variables are presented in Table 2. Additionally, we included bivariate correlations in Table 2. Patients' self‐reported preference for information seeking (*M* = 95.9, SD = 8, Range = [0–100]) and intention to participate in the upcoming consultation (*M* = 5.4, SD = 0.9, Range = [1–6]) had ceiling effects, and because of the variance restriction in these two variables, they are precluded from further analyses.

**Table 2:** Descriptive statistics and bivariate correlations of participation preference with predictor variables

Variable	1	2	3	4	5	6	*n*	*M*	SD
1. Participation preference (API‐dm)	‐						427	44.8	26.5
2. Information preference (API‐is)	.036	‐					433	95.9	8.0
3. Age	−.166**	.114*	‐				434	62.5	13.5
4. Intention to participate	.258**	.152**	.152**	‐			422	5.4	0.9
5. Positive attitudes (PABS‐P)	.383**	.166**	−.016	.305**	‐		426	64.9	14.4
6. Negative attitudes (PABS‐N)	−.317**	−.044	.149**	−.14**	.02	‐	423	55.8	12.8

*Note*: Differences in the number of patients (*n*) are due to missing data.

Abbreviations: API, Autonomy Preference Index; API‐dm, decision‐making subscale of the API; API‐is, information‐seeking subscale of the API; PABS‐N, negative subscale of the Patients' Attitudes and Beliefs Scale; PABS‐P, positive subscale of the Patients' Attitudes and Beliefs Scale.

* *p* ≤ .05;

** *p* ≤ .01.

The primary outcome, patients' participation preference (API‐dm), correlated highest with the two attitudes subscales (positive attitudes: *r* = .383, *p* ≤ .01; negative attitudes *r* = −.317, *p* ≤ .01), with medium effect sizes.^52^ Age correlated negatively with participation preference, with older patients reporting a lower participation preference (*r* = −.166, *p* ≤ .01), corresponding to a small effect size. Furthermore, patients' participation preference was significantly associated with education (η = 0.191, *p* ≤ .01), occupation (η = 0.2, *p* ≤ .01) and living arrangement (η = 0.107, *p* ≤ .05), each with a small effect size.

The associations of patients' gender and type of diagnosis with participation preference were nonsignificant (*p* ≥ .14) and, thus, excluded from the multivariate mediation analysis. Furthermore, we excluded occupation from the mediation analysis due to substantial multicollinearity with age (η = 0.7, *p* ≤ .001).

Based on the results of the bivariate analyses described above, the following variables with a significant association with participation preference were included in the mediation model: age, education and living arrangement as exogenous predictors; positive and negative attitudes as endogenous mediators of the effect and participation preference as the endogenous outcome variable.

### 3.3 Mediating effects of patients' attitudes and beliefs

**Figure 1:**
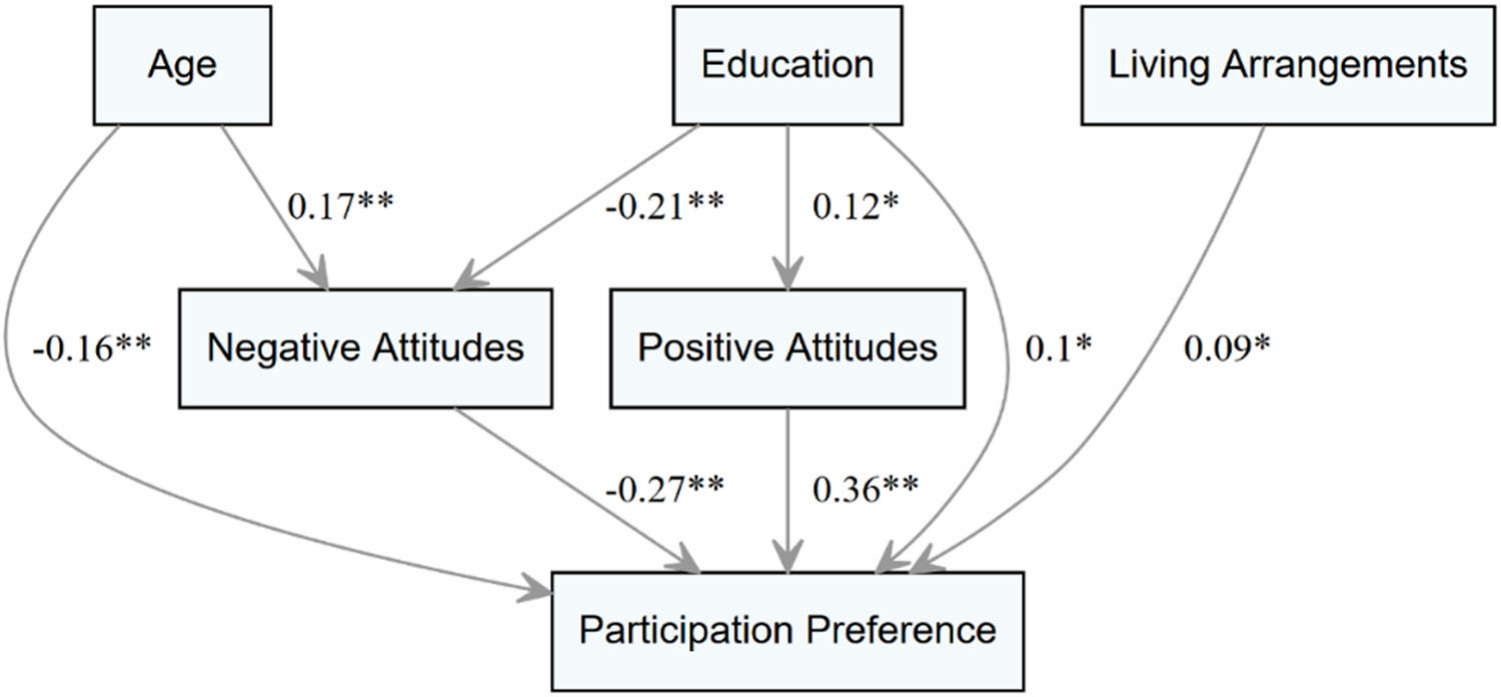
Path model of the mediating effect of attitudes and beliefs. First row: Sociodemographic variables as exogenous predictors (age, education and living arrangement). Second row: Endogenous mediators (positive and negative attitudes and beliefs towards decision‐making). Third row: Primary outcome (participation preference). Paths (i.e., arrows) indicate significant standardized *β* weights. Nonsignificant paths omitted (a nonomitted version is depicted in Supporting Information: Appendix C, Figure C1). * *p* ≤ .05; ** *p* ≤ .001.

The assumptions of multivariate normality for all endogenous variables included in the path analysis (negative attitudes, positive attitudes and participation preference) were examined with the Mardia Test of Skewness (*b1p* = 11.2, *p* = .34) and Mardia Test of Kurtosis (*b2p* = 1.4, *p* = .16)^65^ as well as with the Henze–Zirkler test (HZ = 1.001, *p* = .1). We included patient characteristics which were significantly bivariate associated with participation preference as exogenous predictors in the path analysis (i.e., age, education and living arrangements). Positive and negative attitudes and beliefs were entered into the model as mediators. Since all paths are theoretically plausible, no constraints were set. Thus, the model is fully saturated (no fit characteristics can be reported). The path model is depicted in Figure 1. The model explained 27.8% of the overall variance in our primary outcome participation preference.

We provide an overview of all effects in the path analysis in Table 3. All three exogenous predictor variables (patients' age, education and living arrangements) significantly affected participation preference (Table 3). Higher age (*ß* = −0.05, *p* ≤ .01), a lower level of education (no university degree) (*ß* = −0.2, *p* ≤ .001) and living arrangement (living alone) (*ß* = 0.12, *p* ≤ .01) were associated with lower participation preference.

**Table 3:** Effects in the mediation analysis between sociodemographic variables and participation preference

Type	Effect	Estimate	*SE*	95% CI		β	*z*	*p*
				Lower	Upper			
Age								
Indirect	Age ‐> Neg ‐> API	−0.090	0.029	−0.148	−0.033	−.047	−3.067	.002
Indirect	Age ‐> Pos ‐> API	−0.032	0.036	−0.102	0.038	−.017	−0.896	.370
Direct	Age ‐> API	−0.304	0.080	−0.461	−0.148	−.158	−3.808	.000
Total	Age ‐> API	−0.427	0.085	−0.592	−0.261	−.222	−5.049	.000
Level of Education								
Indirect	Edu ‐> Neg ‐> API	3.049	0.886	1.313	4.786	.056	3.441	.001
Indirect	Edu ‐> Pos ‐> API	2.344	1.047	0.291	4.397	.043	2.238	.025
Direct	Edu ‐> API	5.666	2.383	0.995	10.337	.103	2.377	.017
Total	Edu ‐> API	11.059	2.614	5.935	16.183	.202	4.230	.000
Living arrangements								
Indirect	Liv ‐> Neg ‐> API	0.915	0.834	−0.720	2.549	.014	1.097	.273
Indirect	Liv ‐> Pos ‐> API	1.272	1.090	−0.865	3.409	.020	1.166	.243
Direct	Liv ‐> API	5.455	2.542	0.473	10.437	.086	2.146	.032
Total	Liv ‐> API	7.641	2.917	1.924	13.359	.120	2.619	.009

Abbreviations: API, participation preferences as measured with the Autonomy Preference Index; Edu, level of education; Liv, living arrangements of the patients; Neg, negative attitudes; Pos, positive attitudes.

Negative attitudes partly mediated the effects of age and education on participation preference. Higher age and lower education levels were both associated with more negative attitudes (Figure 1), which significantly predicted lower participation preference (*β* = −.27, *p* ≤ .001). More positive attitudes further mediated the effect of education on participation preference. Last, living arrangements directly affected participation preference but had no significant relationship with positive or negative attitudes.

## 4 CONCLUSION

### 4.1 Discussion

Shared decision making (SDM) is generally recommended in medical consultations, but there are barriers to its successful implementation. Some of these barriers (i.e., attitudes and beliefs) are clearly more accessible to change than others (i.e., age). We examined their combined effects in patients who faced preference‐sensitive decisions in urological consultations. Interestingly, we found that patients' attitudes about their role in the interaction and their personal beliefs about acceptable behaviours in the decision‐making process were strong predictors of patients' participation preference in decision‐making.

With reference to earlier studies, we replicated the association of participation preference and sociodemographic characteristics,^26^ for example, age, education, occupation and living arrangements in urological patients. Specifically, we found that older patients and those who are less educated and live alone have a lower generic participation preference. More importantly, as a noteworthy extension of the existing literature, our data show that patients' attitudes and beliefs mediate their influence on participation preference. This novel finding aligns with recent theoretical considerations suggesting that less educated and older patients may not feel empowered to participate even if they wanted to.^30^, ^41^

Overall, the predictors in our mediation path model explained around 28% of the variance in patients' participation preferences, which corresponds to the strong explanatory power of our model.^52^ In previous research, multiple regressions were mainly used to analyse factors influencing participation preferences and explained between 3% and 21% of the variance.^66^, ^67^, ^68^ While our mediation path model explained a substantial amount of variance and, more importantly, uncovered the mediating effects of attitudes and beliefs, it still leaves room for improvement. Future studies could include other relevant influencing factors, for example, a measure of perceived behavioural control.

Our findings underscore the importance of assessing patients' attitudes and beliefs regarding their role in decision‐making. Instead of assuming that older or less educated patients would not want to participate, we found that these patients are encumbered by negative attitudes and beliefs about their role. To enable and adequately support patient participation, clinicians should challenge patients' negative beliefs (e.g., ‘It would offend my doctor if I were to make my own decision(s)’).

Furthermore, our findings emphasize the general notion that decision‐making is not always rational. Instead, negative emotions (e.g., fear of being labelled as a bad patient^29^, ^33^) can induce avoidance, even if this behaviour incurs costs (see our experimental work; e.g., Pittig et al.^69^). In patients with negative attitudes and paternalistic beliefs, this may hamper patients' expressing their personal needs.

Patients are thought to be especially prone to experiencing power imbalance in the relationship with their physicians when the stakes are high, for example, when they face a life‐threatening disease.^41^ However, we did not find a difference between the oncological and the nononcological subsamples. A possible explanation is that the nononcological patients in our sample presented with complex and long‐lasting medical conditions and consequently faced substantial burdens as well (see Köther et al.^57^). In line with this, it is possible that patients' attitudes, beliefs and participation preferences are not directly associated with illness severity but rather with the perceived burden and impairment associated with it.

In addition, there were significant differences between the oncological and the nononcological subsamples, for example, the urooncological sample contained more male patients, they were on average older and more were retired. Thus, the high homogeneity in this sample and lack of variance could have limited the possibility of finding differences in attitudes and beliefs. This might limit the generalizability to the bigger concept of the influence of illness severity (i.e., life‐threatening or not). However, it does not speak against the generalizability to urological patients, as the described differences in the distribution of gender, age and occupation are typical in urology versus urooncology.

Regarding the information preference of the patients, we found considerable ceiling effects in the API‐is, similar to previous reports.^27^, ^59^, ^60^, ^70^ Unfortunately, the lack of variance obtained by the measurement prevented us from including it in further analysis. However, it is important to recognize that urological and urooncological patients generally have a genuinely high preference for being informed, even when reporting a lower preference for participation. The finding of a universally high preference for information is consistent with studies on other patient groups, for example, patients at the general practitioner^70^ or patients with end‐stage renal disease^71^ or a study with over 5000 older adults who all have a very high desire for information.^72^

Thus, to treat uro(onco)logical patients according to their preferences, they should readily receive information on diagnostics, treatment options and side effects regardless of their preference for participation in the decision‐making process.

### 4.2 Limitations

One limitation of our study is the utilization of the attitudes and beliefs scale as a proxy for a power imbalance. Even though attitudes and beliefs, as measured by the PABS,^55^ were the strongest predictors of participation preference and capture essential aspects of the power imbalance in the patient–physician relationship, such as attitudes towards the behaviour, control and normative beliefs, the questionnaire does miss out on some relevant facets. While we recognize the importance of having a short questionnaire, we argue that the five items on the negative subscale (PABS‐N) are missing some important concepts.

For example, the PABS‐N lacks items that assess perceived dependency on the physician, the avoidance of speaking up or how the patients value having a good relationship with the physician. An additional direct measure of perceived behaviour control, for example, self‐efficacy in the context of medical decision‐making, would be an interesting extension in future studies. Moreover, there is a limitation in the way the PABS was translated. While we did assess the quality of the translation with an independent bilingual speaker, as well as a short pilot with urooncological patients, the validity of the translation could have been improved by further conducting in‐depth cognitive interviews. To address these limitations, we are currently developing a questionnaire that is explicitly designed to capture the construct of power asymmetry in the relationship in broader terms.

Furthermore, generalization from the chosen urological sample to other patient groups maybe not be warranted. Our sample was primarily male and of higher age. While this is expected in a urological sample, it is unclear if our results generalize well to other medical conditions, for example, if the found mediation is only prevalent in primarily male patients. While a patient's gender and primary diagnosis did not have a meaningful effect on participation preference or attitudes and beliefs, it remains an open question if attitudes and beliefs have a mediating effect on patients' participation preference in other cancer types or patient groups.

Finally, structural equation analysis is generally not suitable for identifying causal relationships. However, with respect to the direction of the mediation, it is plausible that age may influence attitudes, whereas the opposite relationship is impossible.

### 4.3 Conclusion and future directions

With increasing international efforts to better implement SDM,^8^, ^73^ it is important to identify barriers that limit patients' ability to participate actively. We found low participation preference in older male patients with less education to be mediated by patients' attitudes and beliefs about their role in decision‐making. However, such patient characteristics cannot be (easily) modified. In accordance with psychological theories, it may be much more promising to empower patients by actively targeting their attitudes and beliefs (see Ajzen and colleagues^43^, ^44^). This may open up new avenues for interventions that prepare patients for the decision‐making process. In SDM, the fear of burdening the physician by asking too many questions^74^ may be amenable to change. Such interventions should aim to redefine perceptions of what constitutes a ‘good patient’. Successful implementation of SDM may require a shift in the power asymmetry.^75^ One way to achieve this may be to explicitly challenge patients' attitudes and beliefs about the inferiority of their role in the decision‐making process.

An obvious first step may be to reassure them that participation will not result in a negative consequence but instead could be a valuable resource for the decision‐making process. Interestingly, small interventions may have a profound impact, as even the most simple instructions have previously shown promise in changing responses to others.^76^ For example, SDM implementation research has previously tried to engage patients, that is, by giving them question prompt lists (e.g., the cancer consultation preparation package^77^ or the AskShareKnow campaign^78^). Such question prompt lists showed some promising first results, as they increased patient activity and led patients to ask more questions.

Yet, it is often difficult to employ such interventions in clinical practice most importantly due to time restraints. Outsourcing these interventions, for example, to online decision aids, may ameliorate this. In urooncology, existing decision aids generally have not addressed patients' attitudes and beliefs,^79^ and thus, they may miss an opportunity to pave the way for SDM. Also, participating in online self‐help groups was shown to reduce negative attitudes in (cancer) patients and should be encouraged by clinicians.^80^ Implementation of SDM in urology and urooncology is especially important,^45^ given that patients, in the course of their treatment, face many preference‐sensitive decisions.^47^, ^48^, ^81^ Research on SDM has documented the positive impact patient participation can have in urology.^82^, ^83^, ^84^ At its core, SDM requires a fundamental shift in power,^85^ which can only be achieved by the active empowerment of patients in urology. Future studies should examine if targeting attitudes and beliefs, for example, in decision aids, holds its promise and helps empower patients in the process of SDM.

It is important to consider that patients' negative attitudes and beliefs and the accompanying fear of being perceived as a ‘bad patient’ may arise from experience. A patient may become quickly disheartened if he/she is willing to participate but experiences a lack of time or responsiveness from the clinicians. In line with the title of a recent SDM intervention study, ‘Changing patients but not physicians is not enough’,^77^ it could be a valuable measure for SDM adaption to also prepare physicians to embrace their patients' willingness to participate in decision‐making.

Finally, our study highlights the importance of patients' attitudes and beliefs regarding the perceived power asymmetry before the consultation for their active participation. Building on that, an interesting path for future studies would be the investigation of the experienced power asymmetry in the medical encounter and the influence on the perceived participation after the encounter.

### 4.4 Practice implications

Our data emphasize the importance of addressing patients' attitudes and beliefs about their patient role in SDM. Targeting attitudes and beliefs can potentially be a valuable intervention to overcome traditional barriers to active participation, such as higher age or lower education. The role of patients' attitudes and beliefs about active participation should be considered in the assessment and targets of future decision aids to empower patients. It may ultimately help to overcome power asymmetry and foster patient‐centred healthcare.

## AUTHOR CONTRIBUTIONS

**Björn Büdenbender**: Methodology; data curation; formal analysis; writing – original draft; writing – review & editing; visualization. **Anja K. Köther**: Data curation; writing – review & editing. **Britta Grüne**: Investigation; data curation. **Maurice S. Michel**: Funding acquisition; resources. **Maximilian C. Kriegmair**: Funding acquisition; conceptualization; resources; supervision. **Georg W. Alpers**: Conceptualization; supervision; writing – review & editing; funding acquisition; resources. All authors approved the final version of the report.

## CONFLICT OF INTEREST

The authors declare no conflict of interest.

## ETHICS STATEMENT

This study was approved by the research ethics Committee II of the medical faculty Mannheim of the University of Heidelberg (MA‐2019‐635N). The authors confirm that all personal identifiers have been removed or disguised so the patients described are not identifiable and cannot be identified through the details of the story.

## Supporting information

Supporting information.

Supporting information.

Supporting information.

## Data Availability

The data and scripts that support the findings of this study will be deposited on MADATA (University of Mannheim, https://madata.bib.uni-mannheim.de/id/eprint/406) Research Data Repository (doi:10.7801/406) and made available by the authors, without undue reservation, to any qualified researcher.
