# Supplementary material for: When attitudes and beliefs get in the way of shared decision‐making: A mediation analysis of participation preference
Source: Health Expect. 2023 Jan 13;26(2):740–51. doi: 10.1111/hex.13699 (PMC10010103; doi:10.1111/hex.13699)
Supplement: Supplementary file 1 — Supporting information. [file HEX-26--s002.docx]

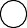

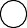

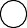

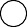


**PABS Fragebogen**


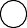
In diesem Fragebogen wird Ihnen eine Reihe an Aussagen präsentiert, zu denen wir Ihre Meinung einholen möchten. Bei den Aussagen geht es um **Ihre Einstellung zu medizinischen Entscheidungen**. Ihre Zustimmung oder Ablehnung können Sie in der Skala von „stimme gar nicht zu“ bis „stimme voll zu“ angeben. Es gibt dabei keine richtigen oder falschen Antworten. Bitte beantworten Sie die Fragen ehrlich.

| stimme gar nicht zu | stimme nicht zu | neutral | stimme zu | stimme voll zu |
| --- | --- | --- | --- | --- |
| 1. Ich weiß nicht genug, um meine eigenen medizinischen Entscheidungen zu treffen. | 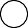 | 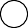 | 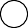 | 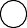 |
| 2. Ärzte sind nicht perfekt, deshalb ist es  wichtig, dass ich in meine medizinischen 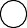 Entscheidungen einbezogen werde. | 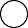 | 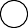 | 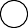 | 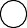 |
| 3. Mein Arzt würde sich angegriffen fühlen,  wenn ich meine eigene/n Entscheidung/en 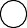 treffen würde. | 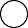 | 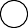 | 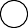 | 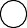 |
| 4. Ich würde lieber viele Entscheidungsmöglichkeiten haben was das Beste für meine Gesundheit ist, als den Arzt die Entscheidung für mich treffen zu lassen. | 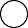 | 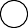 | 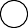 | 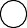 |
| 5. Ich habe das Recht meine eigenen  medizinischen Entscheidungen zu treffen, 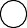 schließlich ist es mein Leben. | 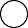 | 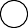 | 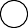 | 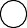 |
| 6. Wenn ich die Entscheidung für die Behandlung treffe, ist es meine Schuld, wenn sich herausstellt, dass es eine schlechte Wahl wahr. | 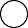 | 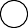 | 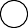 | 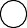 |
| 7. Ich hätte weniger Vertrauen in meinen Arzt,  wenn er mir nicht sagen würde, was ich tun 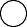 soll. | 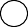 | 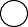 | 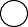 | 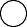 |
| 8. Ich wäre töricht, meinem Arzt völlig zu vertrauen. | 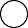 | 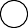 | 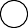 | 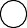 |
| 9. Ich treffe oft miese Entscheidungen. 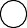 | 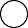 | 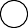 | 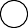 | 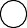 |
| 10. Ich neige dazu, eine zweite Meinung einzuholen, wenn ich mit einer schwerwiegenden medizinischen Entscheidung konfrontiert werde. | 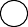 | 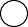 | 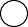 | 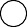 |
| 11. Meine eigenen medizinischen  Entscheidungen zu treffen ermöglicht es mir 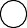 Kontrolle über meine Gesundheit zu haben | 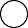 | 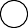 | 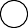 | 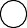 |
| 12. Die Mitwirkung an meinen medizinischen Entscheidungen ist gut für meine 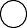 Gesundheit. | 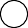 | 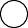 | 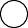 | 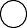 |
