# Supplementary material for: When attitudes and beliefs get in the way of shared decision‐making: A mediation analysis of participation preference
Source: Health Expect. 2023 Jan 13;26(2):740–51. doi: 10.1111/hex.13699 (PMC10010103; doi:10.1111/hex.13699)
Supplement: Supplementary file 2 — Supporting information. [file HEX-26--s001.docx]

**Appendix B – List of R-Packages for all Analyses**

| **Packagename** | **Version** | **Maintainer** | **Citation** |
| --- | --- | --- | --- |
| **DiagrammeR** | 1.0.6.1 | Richard Iannone <riannone@me.com> | Iannone, R. (2020). *DiagrammeR: Graph/Network Visualization*. https://github.com/rich-iannone/DiagrammeR |
| **haven** | 2.3.1 | Hadley Wickham <hadley@rstudio.com> | Wickham, H., & Miller, E. (2020). haven: Import and Export SPSS, Stata and SAS Files. https://cran.r-project.org/package=haven |
| **MVN** | 5.8 | Selcuk Korkmaz <selcukorkmaz@gmail.com> | Korkmaz, S., Goksuluk, D., & Zararsiz, G. (2019). MVN: Multivariate Normality Tests. https://cran.r-project.org/package=MVN |
| **lavaanPlot** | 0.5.1 | Alex Lishinski <alexlishinski@gmail.com> | Lishinski, A. (2018). lavaanPlot: Path Diagrams for Lavaan Models via DiagrammeR. https://github.com/alishinski/lavaanPlot |
| **webshot** | 0.5.2 | Winston Chang <winston@rstudio.com> | Chang, W. (2019). webshot: Take Screenshots of Web Pages. https://github.com/wch/webshot/ |
| **lavaan** | 0.6.7 | Yves Rosseel <Yves.Rosseel@UGent.be> | Rosseel, Y., Jorgensen, T. D., & Rockwood, N. (2020). lavaan: Latent Variable Analysis. http://lavaan.org |
| **labelled** | 2.7.0 | Joseph Larmarange <joseph@larmarange.net> | Larmarange, J. (2020). labelled: Manipulating Labelled Data. http://larmarange.github.io/labelled/ |
| **datscience** | 0.1.0 | Björn Büdenbender <buedenbender@uni-mannheim.de> | https://buedenbender.github. io/datscience/index.html |
| **knitr** | 1.30 | Yihui Xie <xie@yihui.name> | Xie, Y. (2020). knitr: A General-Purpose Package for Dynamic Report Generation in R. https://yihui.org/knitr/ |
| **ggpubr** | 0.4.0 | Alboukadel Kassambara <alboukadel.kassambara@gmail.com> | Kassambara, A. (2020a). ggpubr: ggplot2 Based Publication Ready Plots. https://rpkgs.datanovia.com/ggpubr/ |
| **ggplot2** | 3.3.2 | Thomas Lin Pedersen <thomas.pedersen@rstudio.com> | Wickham, H., Chang, W., Henry, L., Pedersen, T. L., Takahashi, K., Wilke, C., Woo, K., Yutani, H., & Dunnington, D. (2020). ggplot2: Create Elegant Data Visualisations Using the Grammar of Graphics. https://cran.r-project.org/package=ggplot2 |
| **psych** | 2.0.9 | William Revelle <revelle@northwestern.edu> | Revelle, W. (2020). psych: Procedures for Psychological, Psychometric, and Personality Research. https://personality-project.org/r/psych/ |
| **tidyr** | 1.1.2 | Hadley Wickham <hadley@rstudio.com> | Wickham, H. (2020b). tidyr: Tidy Messy Data. https://cran.r-project.org/package=tidyr |
| **dplyr** | 1.0.2 | Hadley Wickham <hadley@rstudio.com> | Wickham, H., François, R., Henry, L., & Müller, K. (2020). dplyr: A Grammar of Data Manipulation. https://cran.r-project.org/package=dplyr |
| **pacman** | 0.5.1 | Tyler Rinker <tyler.rinker@gmail.com> | Rinker, T., & Kurkiewicz, D. (2019). pacman: Package Management Tool. https://github.com/trinker/pacman |
