# Supplementary material for: When attitudes and beliefs get in the way of shared decision‐making: A mediation analysis of participation preference
Source: Health Expect. 2023 Jan 13;26(2):740–51. doi: 10.1111/hex.13699 (PMC10010103; doi:10.1111/hex.13699)
Supplement: Supplementary file 3 — Supporting information. [file HEX-26--s003.docx]

# Appendix C – Full Path Model

For the path model in Figure 1, we chose to omit non-significant paths for increased readability. Figure C1 shows the same path model of the proposed mediation (i.e., sociodemographic variables - attitudes and beliefs - participation preference), including all non-significant paths.

**Figure C1**

Full Path Model of the Mediating Effect of Attitudes and Beliefs. First row: Sociodemographic variables as exogenous predictors (age, education, and living arrangement). Second row: endogenous mediators (positive and negative attitudes and beliefs towards decision-making). Third row: primary outcome (participation preference). Paths are depicted as arrows. Asterisks indicate significance.


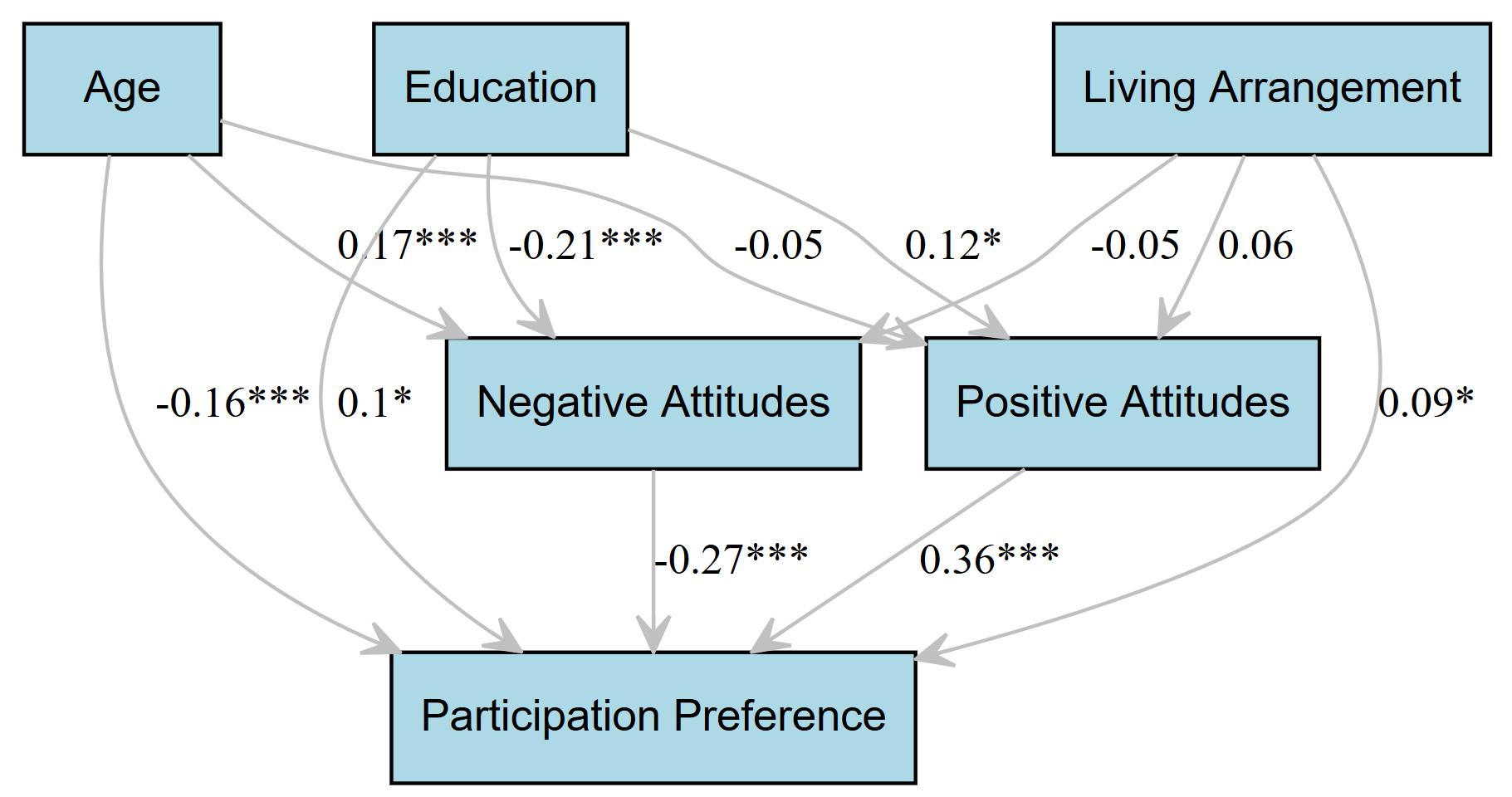


*Note.* * *p* ≤ 0.05 , *** *p* ≤ .001.
